# Editorial: Women in psychiatry 2022: psychological therapies

**DOI:** 10.3389/fpsyt.2023.1234952

**Published:** 2023-08-01

**Authors:** Tihana Jendričko

**Affiliations:** Faculty of Law, University Psychiatric Hospital Vrapče, Zagreb, Croatia

**Keywords:** psychological therapies, early intervention, mental health, prevention, psychological therapy

For many decades, the effectiveness of psychological treatments has been an interesting topic for scientific assessment. Several studies concluded that patients receiving psychological treatment improved at the end of the treatment compared to those who did not receive such treatment. At the same time, we might say that there is a lack of consistent scientific assessment of various psychotherapeutic approaches and interventions. Psychological interventions are an important part of mental health treatment because they can minimize the harm caused by the mental disorder while also promoting age-appropriate psychological development and social competence. Psychological interventions may also improve the patient's and relatives' commitment to overall treatment and maintain or improve the patient's level of psychological and social functioning while being less expensive and having no side effects.

This editorial highlights five excellent articles focusing on various psychological therapies and how they can be implemented for providing effective treatment, reducing stress, and improving the quality of life. Gergov et al. in their systematic review present the research evidence from clinical trials on the effectiveness of psychological interventions for treating young people with psychotic disorders. They highlighted that psychotherapy not only reduces symptoms but also provides an important and added value in the treatment of psychological functions and relational functioning. Tenore et al. introduce imagery rescripting (ImR) as a therapeutic technique that aims at reducing the distress associated with negative memories of early aversive experiences. They researched the effectiveness of a group ImR intervention via telehealth, and its effectiveness in reducing dysfunctional beliefs and changing participants' affective state. Freidl et al. studied positive changes in the cognitively mediated perception of quality of life, despite possibly remaining clinical symptoms. Quality of life is an important indicator of response to psychotherapy, especially since mental disorders generally have a detrimental effect on it. For certain patients, more targeted treatment should be engaged. Takeuchi et al. discussed the problem of identifying mothers with depressive symptoms. In a large-scale survey of mothers and children, authors developed the multidimensional physical scale (MDPS) as a useful screening test for assessing mild depression by the physical aspects of mothers from the Kampo medicine perspective. Töbelmann et al. researched Yoga-based Group Intervention specifically developed for the treatment of in-patients with schizophrenia spectrum disorders. This low-cost group intervention could increase wellbeing and substantial symptom improvements. This intervention has an important destigmatizing and normalizing approach. One of the important advances is inclusivity while tailoring the practice specifically to the needs of the targeted patients, as it can also play an important part in patients' improvement by reducing feelings of psychological stress, isolation, shame, and stigmatization.

In conclusion, the National Institute for Health and Care Excellence (NICE), as well as other well-known clinical guidelines and research that make evidence-based recommendations on a wide range of health, public health, and social care topics, include psychological interventions in their recommendations. We believe that the studies covered in this Editorial will provide and enrich our perspectives regarding the valuable implementation of various, especially early, psychological interventions that can be helpful not only in treatment but also in the prevention of mental health disorders.

## Author contributions

The author confirms being the sole contributor of this work and has approved it for publication.

